# Curcumin as a Promising Antibacterial Agent: Effects on Metabolism and Biofilm Formation in* S. mutans*

**DOI:** 10.1155/2018/4508709

**Published:** 2018-02-28

**Authors:** Bingchun Li, Xinlong Li, Huancai Lin, Yan Zhou

**Affiliations:** ^1^Department of Preventive Dentistry, Guanghua School of Stomatology, Sun Yat-sen University, 56 Ling Yuan Road West, Guangzhou 510055, China; ^2^Guangdong Provincial Key Laboratory of Stomatology, Sun Yat-sen University, Guangzhou 510055, China

## Abstract

*Streptococcus mutans (S. mutans)* has been proved to be the main aetiological factor in dental caries. Curcumin, a natural product, has been shown to exhibit therapeutic antibacterial activity, suggesting that curcumin may be of clinical interest. The objective of this study is to evaluate the inhibitory effects of curcumin on metabolism and biofilm formation in* S. mutans* using a vitro biofilm model in an artificial oral environment.* S. mutans* biofilms were treated with varying concentrations of curcumin. The biofilm metabolism and biofilm biomass were assessed by the 3-(4,5-dimethylthiazol-2-yl)-2,5-diphenyl tetrazolium bromide assay and the crystal violet assay. Confocal laser scanning microscopy was used to analyse the composition and extracellular polysaccharide content of* S. mutans* biofilm after curcumin treatment. The biofilm structure was evaluated using a scanning electron microscope. The gene expression of virulence-related factors was assessed by real-time PCR. The antibiofilm effect of curcumin was compared with that of chlorhexidine. The sessile minimum inhibitory concentration (SMIC_50%_) of curcumin against* S. mutans* biofilm was 500 *μ*M. Curcumin reduced the biofilm metabolism from 5 min to 24 h. Curcumin inhibited the quantity of live bacteria and total bacteria in both the short term (5 min) and the long term. Moreover, curcumin decreased the production of extracellular polysaccharide in the short term. The expression of genes related to extracellular polysaccharide synthesis, carbohydrate metabolism, adherence, and the two-component transduction system decreased after curcumin treatment. The chlorhexidine-treated group showed similar results. We speculate that curcumin has the capacity to be developed as an alternative agent with the potential to reduce the pathogenic traits of* S. mutans* biofilm.

## 1. Introduction

Dental caries, one of the most prevalent infectious diseases worldwide, is a biofilm-mediated, sugar-driven, multifactorial, dynamic disease [1]. Although the pathobiology of dental caries is complicated, it is widely recognized that the formation of dental plaque biofilm is one of the important causes of dental caries [2, 3]. Biofilm formation creates an anaerobic and acidic environment that results in the formation and development of caries [4]. Therefore, the eradication of dental biofilm is an effective method of controlling caries.

*Streptococcus mutans (S. mutans)* has been proved to be the main aetiological factor in dental caries [5, 6]. By adhering to solid surfaces,* S. mutans *can colonize the oral cavity and form a bacterial biofilm [7]. Dental biofilms are microbial aggregates encased in a self-produced extracellular polymer matrix. The phenotype of the bacteria involved in biofilm formation is quite different from the phenotype in the planktonic state [8]. The bacteria in a dental biofilm are far more resistant to unfavourable growth conditions, such as biocides and hydrodynamic shear forces [9, 10]. The extracellular polysaccharide (EPS) produced by* S. mutans *through glycosyltransferases (Gtfs) has been shown to form the matrix of the biofilm [11]. The EPS mediates the irreversible adherence between bacteria to form a high-cell-density biofilm [12], which increases the bacterial resistance to antibiotics.

Currently, many natural products have been considered as alternative or adjunctive anticaries therapies, particularly botanically derived molecules, which offer advantages over synthetic derivatives due to their natural evolution and diminished likelihood of resistance [13]. Curcumin, the major constituent of* Curcuma longa* L. or turmeric, has been confirmed as a potential therapeutic antibacterial agent [14]. Curcumin has been reported to inhibit various bacteria:* Staphylococcus aureus, Salmonella paratyphi, Trichophyton gypseum*, and* Mycobacterium tuberculosis* [14]. Recent publications have also reported that curcumin is active against a plethora of drug-resistant bacterial strains [14].

Studies have shown the effects of curcumin on oral bacteria associated with dental disease. Song et al. [15] found that curcumin could significantly inhibit the adhesiveness of* S. mutans* by its effects on collagen and fibronectin. Hu et al. [16, 17] reported that curcumin is an* S. mutans* sortaseA inhibitor and has promising anticaries characteristics based on an antiadhesion-mediated mechanism. Manoil et al. [18] found that blue light-activated curcumin can photoinactivate planktonic* S. mutans*, but the effect on* S. mutans* biofilm was poor. These studies proposed the potential use of curcumin as an antibacterial agent and underlined the need to explore the mechanism by which curcumin acts on oral bacteria.

Our research aims to explore the effects of curcumin on the biofilm formed by* S. mutans* and compares the results with the effects of chlorhexidine. The study contributes to the possibility of a natural medicine with fewer side effects and stronger antibacterial effects, thereby promoting the wider clinical application of curcumin.

## 2. Materials and Methods

### 2.1. Bacterial Strain and Growth Conditions

*S. mutans* strain UA159 (ATCC700610) was provided by the Guangdong Microbial Culture Collection Center. The* S. mutans* growth in brain-heart infusion (BHI, Difco, Detroit, MI, USA) broth medium was measured. For biofilm formation, an overnight culture of bacteria was adjusted to OD = 1.0 (1*∗*10^8^ CFU/ml). The adjusted bacteria were inoculated into 96-well flat-bottom plates. The medium was 1% BHIS, that is, BHI with the addition of 1% (wt/vol) sucrose. The bacteria were grown at 37°C under anaerobic conditions (5% CO_2_, 10% H_2_, 85% N_2_).

### 2.2. Minimum Inhibitory Concentration Assay

The minimum inhibitory concentration (MIC) of curcumin against planktonic* S. mutans* was determined by microdilution methods following the Clinical Laboratory Standards Institute (CLSI) procedure [19], with some modifications as described below. Curcumin from a 250 mM stock in dimethyl sulfoxide (DMSO) was diluted in BHI. The resulting solution was then serially diluted in sterile BHI broth in a 96-well round-bottom plate to obtain 2000, 1000, 500, 250, 125, 62.5, and 31.25 *μ*M curcumin in BHI culture medium. The corresponded concentrations of DMSO were used as control. Each well contained 100 *μ*l of serially diluted curcumin, to which 100 *μ*l of an overnight culture of* S. mutans *was added before incubation at 37°C under anaerobic conditions (5% CO_2_, 10% H_2_, 85% N_2_) for 24 h. Three parallel samples were prepared at each concentration, and BHI without curcumin was used as a control. The MIC was defined as the lowest concentration at which no visible bacteria grew in the broth.

The sessile minimum inhibitory concentration (SMIC) of curcumin against biofilm* S. mutans *was determined by the 3-(4,5-dimethylthiazol-2-yl)-2,5-diphenyl tetrazolium bromide (MTT) assay. Overnight* S. mutans* UA159 cultures were adjusted with 1% BHIS to an initial optical density at 600 of 0.1 (approximately 10^7^ CFU/ml), and equal volumes (200 *μ*l) of diluted bacteria were added to 96-well plates and incubated for 24 h at 37°C in a humidified atmosphere containing 5% CO_2_. After 24 h, the planktonic bacteria were removed carefully, and the biofilms formed on the plates were washed with sterile phosphate buffered saline (PBS). The stock solution of curcumin was diluted in BHIS broth to concentrations ranging from 15.625 *μ*M to 1000 *μ*M and added to the test wells. The corresponded concentrations of DMSO were used as control. After 24 h of incubation, the BHIS broth with curcumin was removed carefully, and the biofilms formed on the plates were washed again with sterile PBS. To quantify the biofilms, 200 *μ*l of 0.5 mg/ml MTT was added to each well and incubated at 37°C in the dark in a humidified atmosphere for 3 h. Then, the supernatants were removed, and 100 *μ*l of 100% DMSO was added to each well. For each well, the absorbance at 570 nm was recorded using a microplate reader. The percentage of biofilm viability was calculated as follows: (absorbance of treated group/absorbance of control group) × 100% [22].

### 2.3. Assays of the Viability and Biomass of* S. mutans* Biofilm

The biofilm viability and biomass were tested at 5 min, 10 min, 15 min, 30 min, 60 min, and 24 h using the MTT assay and the crystal violet (CV) assay [20]. The SMIC_50%_ concentration of curcumin was selected for the assay, and 0.12% (w/t) chlorhexidine was used in the experiment for comparison and the 0.12% (w/t) water was used as control. For the CV assay, the contents of the microplate were removed after incubation, and the wells were washed with PBS, fixed with 95% methanol, washed again, and stained with 0.1% (wt/vol) crystal violet solution for 15 min at room temperature. Subsequently, the microplates were vigorously tapped on napkins to remove any excess liquid and air-dried. The remaining CV was dissolved in 100 *μ*l of 100% ethanol for 15 min at room temperature, and, finally, 75 *μ*l from each sample was transferred to a new 96-well plate, and the extract was read at 600 nm in a spectrophotometer [20, 21].

### 2.4. Analysis of the Bacteria Composition and Extracellular Polysaccharide (EPS) of* S. mutans* Biofilm

#### 2.4.1. Analysis of Live and Dead Bacteria by Confocal Laser Scanning Microscopy (CLSM)

As described previously, a 24 h biofilm of* S. mutans* was formed on a 15 mm confocal dish at 37°C in a humidified atmosphere containing 5% CO_2_. The planktonic bacteria were removed and the biofilm was washed twice with sterile PBS. The biofilm was treated with 500 *μ*M curcumin, 0.12% chlorhexidine, or 1% BHIS as a control for 5 min or 24 h. After treatment, the biofilm was stained with L-7012 LIVE/DEAD®* BacLight*™ Bacterial Viability Kits (Molecular Probes, Eugene, OR, USA). The live/dead stain, stored at −20°C, was warmed to room temperature and centrifuged before use. The staining solution included two components, SYTO9 and propidium iodide, which were mixed in equal quantities and applied to the dish for 15 min. The excitation wavelengths for SYTO9 and propidium iodide are 488 and 543 nm. The biovolumes of live and dead cells were quantified from the entire stack using COMSTAT image-processing software. The biovolume is defined as the volume of the biomass (*μ*m^3^) divided by the area of the substratum (confocal dish) (*μ*m^2^) [22].

#### 2.4.2. Analysis of EPS by CLSM

As described previously, a 24 h biofilm of* S. mutans* was grown in 15-mm confocal dishes, protected from light, with 1 *μ*M Alexa Fluor 647® red fluorescent dye (Invitrogen Corp., Carlsbad, CA, USA) to label the EPS [23]. After incubation for 24 h at 37°C in a humidified atmosphere containing 5% CO_2_, treatments consisting of 500 *μ*M curcumin, 0.12% chlorhexidine, and 1% BHIS as a control were added to separate dishes. Then, after incubation for 5 min or 24 h, the biofilms were gently washed with normal saline and incubated with 1 *μ*M SYTO9 green fluorescent dye at room temperature for 15 min to label the live bacteria. The image collection gates were set to 655–690 nm for Alexa Fluor 647 and 495–515 nm for SYTO9. Images were obtained by CLSM and analysed using the image-processing software COMSTAT [24].

### 2.5. Scanning Electron Microscopy (SEM)

*S. mutans* 24 h biofilm was incubated in 1% BHIS medium with or without 500 *μ*M curcumin or 0.12% chlorhexidine at 37°C for 5 min or 24 h. Next, the* S. mutans* biofilms were washed twice with PBS, fixed with 2.5% glutaraldehyde overnight at 4°C, and washed with PBS. After dehydration by an alcohol gradient (30%, 50%, 70%, 80%, 85%, 90%, 95%, and 100%), the biofilm was dried in a desiccator and sputter-coated with gold. Then, the sample were examined at 2000x, 5000x, and 10,000x magnification by SEM [23, 24].

### 2.6. Assay of Gene Expression by Real-Time PCR

Biofilms grown for 24 h were treated with drugs for 5 min or 24 h. The biofilms were harvested by centrifugation at 12,000 rpm for 5 min, and the total RNA was isolated by ultrasonic crushing and using the RNeasy Mini Kit (QIAGEN, Valencia, CA, USA). The total RNA concentration and purity were determined using a NanoDrop 2000 spectrophotometer (Thermo Fisher Scientific, Pittsburgh, PA, USA). The reverse transcription of the total RNA and quantitative real-time PCR were performed similarl to the procedures described in previous studies [23]. The primer sequences were based on the previous literature and are listed in Table 1 [17, 24–27]. The 2^−ΔΔCt^ method was used to calculate the gene expression fold changes in* S. mutans*.

### 2.7. Statistical Analysis

Each experiment was independently repeated at least three times. GraphPad Prism version 5.04 (GraphPad Software, San Diego, CA) was used to assess the data. The differences between the experimental group and the untreated control group were statistically analysed using the SPSS 17.0 software. The data were assessed to determine whether they were normally distributed. One-way analysis of variance (ANOVA) and Tukey's test were performed for multiple groups. The unpaired *t*-test was used for two groups. The significance level of the *P* value was <0.05.

## 3. Results

### 3.1. Determination of the MIC of Curcumin against Planktonic and Biofilm* S. mutans*

**Figure 1:**
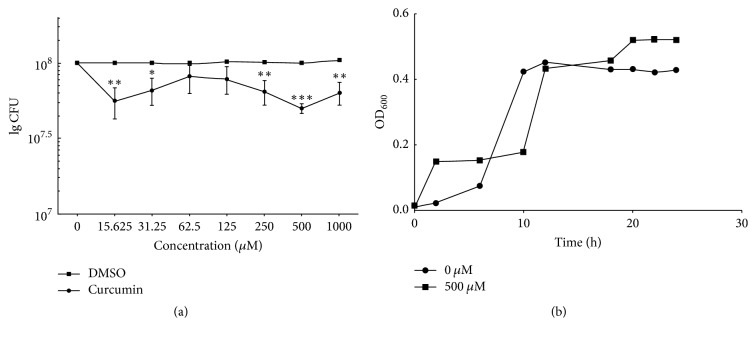
(a) Antibiofilm effects of different concentrations of curcumin and the correspondent concentrations of DMSO on* S. mutans *biofilm. The bacteria were inoculated in a 96-well microtiter plate containing 1% BHIS medium to form a 24 h biofilm, then washed with PBS, and incubated in 1% BHIS with different concentrations of curcumin (^*∗*^*P* < 0.05; ^*∗∗*^*P* < 0.01; ^*∗∗∗*^*P* < 0.001). After 24 h of incubation, the Colony-Forming Units (CFU) of lived bacteria in biofilm were evaluated by the MTT assay. (b) Effect of SMIC_50_ curcumin on the growth of* S. mutans*.

The results showed that curcumin inhibited the growth of planktonic* S. mutans *with an MIC of 125 *μ*M. After treatment with concentrations higher than 125 *μ*M, no visible* S. mutans* growth was observed. The MTT assay showed that, after treatment with serial dilutions of curcumin, the biofilm viability decreased significantly, by 50%, at 500 *μ*M. The DMSO has no effect on viability of biofilm (Figure 1(a)). The growth curve at concentrations of 500 *μ*M and for the control showed similar trends. The bacteria in both cases reached the stationary phase at 12 h (Figure 1(b)). The variation in biofilm biomass between 500 *μ*M and 0 was independent of the growth kinetics. Therefore, a concentration of 500 *μ*M was defined as causing 50% inhibition (SMIC_50%_) and selected for the next study.

### 3.2. Inhibition of the Metabolism of Biofilm Formation by Curcumin

**Figure 2:**
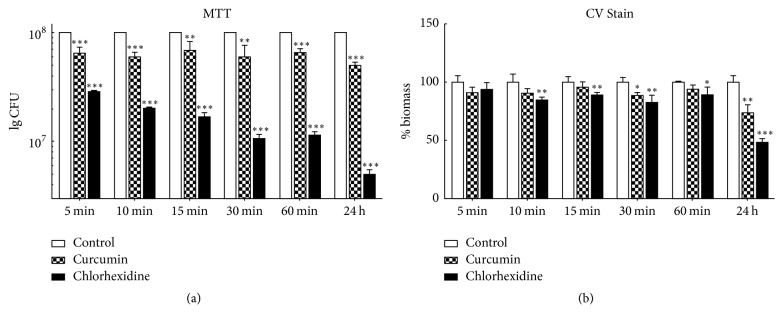
Antibiofilm effect of different curcumin exposure times on* S. mutans *biofilm. A 24 h biofilm was incubated in curcumin at the SMIC_50_ for different times. The Colony-Forming Units (CFU) of lived bacteria in biofilm were evaluated by the MTT assay (a), and the percentage of biofilm biomass was evaluated by the crystal violet (CV) assay (b). The data represent the mean ± SD of three independent tests. The asterisks (*∗*) indicate significant differences (^*∗*^*P* < 0.05, ^*∗∗*^*P* < 0.01, ^*∗∗∗*^*P* < 0.001).

Both MTT and CV assays were conducted to evaluate the alteration in relevant biofilm characteristics of* S. mutans* under the influence of curcumin. In addition, 0.12% chlorhexidine treatment was used to provide a comparison group. The results of the MTT assay showed that curcumin decreased the biofilm metabolism at both 5 min and 24 h compared to that of untreated biofilm (*P* < 0.001, Figure 2(a)). Chlorhexidine treatment produced the same trend as curcumin, and the biofilm metabolism decreased significantly more than for curcumin (*P* < 0.001, Figure 2(a)). The CV assay showed that curcumin and chlorhexidine both resulted in reduced* S. mutans* biomass at 24 h. Chlorhexidine resulted in a significantly greater decrease than curcumin (*P* < 0.001, Figure 2(b)). As curcumin decreased the metabolism of the* S. mutans* biofilm in a short time (5 min), we next compared the short-term (5 min) and long-term (24 h) effects of curcumin on* S. mutans* biofilm.

### 3.3. Evaluation of the Effect on the Live/Dead Bacteria Ratio in* S. mutans *Biofilm

**Figure 3:**
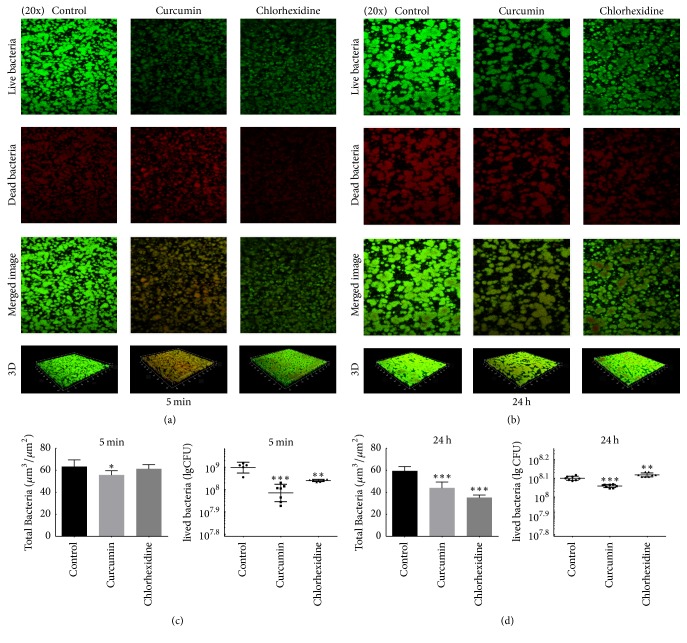
CLSM imaging of* S. mutans* biofilm grown in 1% BHIS. After 24 h of growth, the biofilm was treated with 1% BHIS (control), 500 *μ*M curcumin, or 0.12% chlorhexidine for 5 min (a) and 24 h (b). Each micrograph represents 4 optical sections: green representing live bacteria, red representing dead bacteria, combined green and red from two channel images, and three-dimensional reconstructions of the control biofilm without any treatment, the 500 *μ*M curcumin-treated biofilm, and the 0.12% chlorhexidine-treated biofilm. The total bacteria biomass and the Colony-Forming Units (CFU) of lived bacteria are quantified in (c) and (d). The data represent the mean ± SD. The asterisks (*∗*) indicate significant differences (^*∗*^*P* < 0.05, ^*∗∗*^*P* < 0.01, ^*∗∗∗*^*P* < 0.001).

The effects of curcumin and chlorhexidine on the live/dead bacteria ratio in* S. mutans* biofilm were assayed by CLSM (Figure 3). In the confocal micrographs, green florescence indicates lived cells, while red fluorescence indicates dead cells. The images reflect different green and red florescence intensities from those in the control group. Lower green-stained density than in the control group was observed in both treated groups at 5 min and 24 h (Figures 3(a) and 3(b)). At 5 min, the live bacteria in the curcumin group and chlorhexidine group were reduced, while the total bacteria in the curcumin group were reduced (Figure 3(c)). At 24 h, the live bacteria in the curcumin group were reduced, while the total bacteria were reduced in both groups (Figure 3(d)).

### 3.4. Evaluation of the Effect on the EPS of* S. mutans *Biofilm

**Figure 4:**
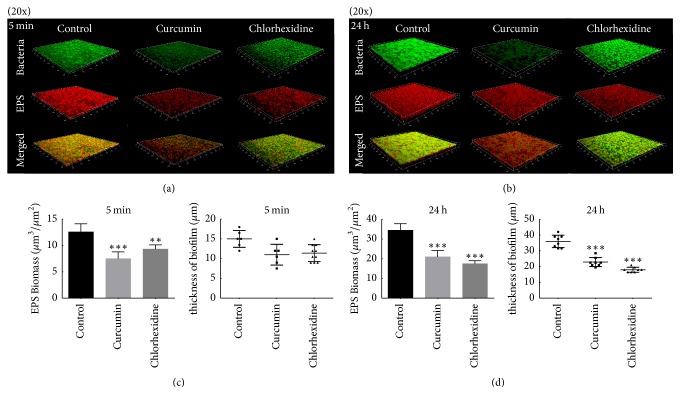
CLSM images of* S. mutans *biofilm. (a, b) Three-dimensional reconstructions of the untreated (control) biofilm, the 500 *μ*M curcumin-treated biofilm, and the 0.12% chlorhexidine-treated biofilm at 5 min (a) and 24 h (b). EPS was labelled in red (Alexa Fluor 647), bacterial cells were labelled in green (SYTO9), and red and green superimposed appear as yellow. Images were obtained at 20x magnification. (c) Image representing the volume of EPS, calculated according to 5 random sites of each sample, repeated three times. (d) Change in biofilm thickness at 5 min and 24 h, respectively, calculated from data obtained from fluorescence CLSM. Data are presented as the mean ± SD. The asterisks (*∗*) indicate significant differences (^*∗∗*^*P* < 0.01, ^*∗∗∗*^*P* < 0.001).

**Figure 5:**
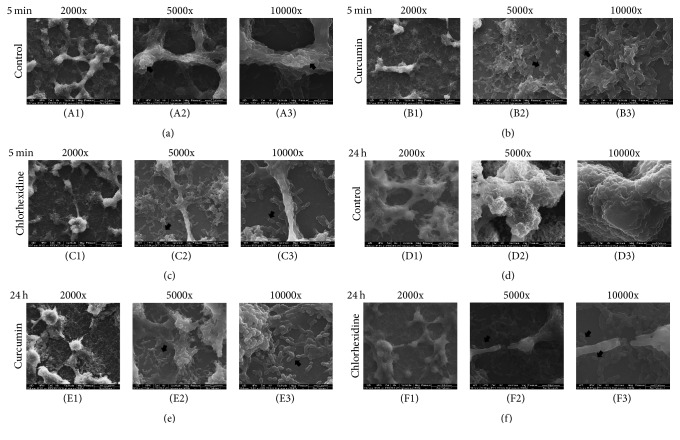
Morphological characteristics of* S. mutans *biofilm treated with or without drugs. Representative SEM images of 24 h* S. mutans *biofilm grown in curcumin for 5 min (b) and 24 h (e) and grown in chlorhexidine for 5 min (c) and 24 h (f). The image of 24 h* S. mutans* biofilm grown in 1% BHIS for 5 min (a) and 24 h (d) as a control. Magnifications of 2000x, 5000x, and 10,000x are shown for each condition. The black arrows highlight the EPS of* S. mutans, *which was markedly reduced.

The three-dimensional reconstruction images obtained from CLSM of the* S. mutans* biofilm (Figures 4(a) and 4(c)) confirmed that the EPS decreased with both curcumin and chlorhexidine treatment after 5 min (*P* < 0.05), and this trend continued at 24 h. The thickness of the biofilm showed no difference among the three groups at 5 min but was visibly reduced at 24 h (Figures 4(b) and 4(d)). The SEM image confirmed the change in EPS (Figure 5). Treatment with curcumin or chlorhexidine was able to reduce the overall quantity of* S. mutans* EPS compared to the untreated control, as indicated by arrows. Furthermore, long-term treatment (24 h) resulted in a more obvious reduction than short-term treatment (5 min).

### 3.5. Evaluation of the Effect on the mRNA Levels of* S. mutans* Biofilm Virulence Factors

**Figure 6:**
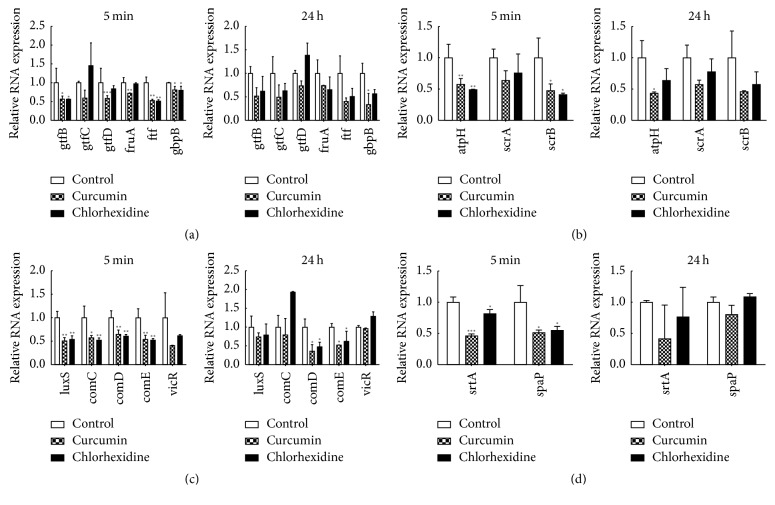
Results of qRT-PCR to examine the gene expression of different virulence systems in* S. mutans* UA159. (a) EPS synthesis system; (b) carbohydrate metabolism system; (c) quorum-sensing system; (d) two-component transduction system. All targets were amplified using primers. Different gene expression levels were normalized to the level of 16sRNA gene transcripts. Data are presented as the mean ± SD. The asterisks (*∗*) indicate significant differences (^*∗*^*P* < 0.05, ^*∗∗*^*P* < 0.01, ^*∗∗∗*^*P* < 0.001).

Compared with the levels observed in* S. mutan*s biofilms grown in 1% BHIS, the expression levels of gtfB, gtfC, and gtfD in* S. mutans* biofilms exposed to 500 *μ*M curcumin for 5 min were significantly decreased by 0.57-, 0.59-, and 0.59-fold, respectively. At 24 h, the expression levels of gtfB, gtfC, and gtfD in* S. mutans *were also decreased, and there was no significant difference from the levels at 5 min. Meanwhile, the expression of gtfB, gtfC, and gtfD was also downregulated under the influence of 0.12% chlorhexidine for 5 min and 24 h; there was no significant difference between two time points (Figure 6(a)).

In Figure 6(b), we observed no significant difference in the expression of scrA at either 5 min or 24 h. However, the expression of atpH and scrB decreased in the presence of curcumin (*P* < 0.05). The expression of atpH decreased further over time. In contrast, the expression of scrB increased over time.

The entire set of virulence genes involved in quorum-sensing signalling mechanisms was found to be downregulated after treatment with curcumin. Treatment with curcumin for 5 min repressed the expression levels of luxS, comC, comD, and comE to 50.7%, 57.4%, 64.8%, and 54.1%, respectively, as compared to the control values. Likewise, chlorhexidine repressed the gene expression levels to 50.1%, 52.3%, 60.7%, and 52.3%, respectively. After treatment with the drugs for 24 h, only the expression of comDE showed a significant difference.

The expression levels of srtA and spaP in the biofilms treated with curcumin for 5 min were significantly downregulated by approximately onefold compared with the levels in untreated biofilm, and the same tendency was shown by chlorhexidine (Figure 6(d)). After 24 h of treatment with either drug, the expression levels of srtA and spaP showed no significant difference.

## 4. Discussion

Dental caries, a common infectious disease worldwide, is caused by biofilm known as dental plaque that result from the adhesion of bacteria to tooth surfaces. The effective eradication of cariogenic biofilm and the pathogenic microorganisms within is the key way to prevent tooth decay [28].* S. mutans *is the main aetiological microorganism involved in biofilm [29]. In this study, we used curcumin, a natural food-grade product, to control the formation of* S. mutans *biofilm.

Previous studies have shown that curcumin could inhibit the viability of* S. mutans* with an MIC value of 125–175 *μ*M [17]. Similar MIC results were obtained in this study, but the SMIC values here were much higher than the MIC. As is well known, it is more difficult to inhibit the viability of* S. mutans *biofilm than planktonic bacteria because the planktonic bacteria are more susceptible to the effects of commonly used antimicrobial drugs than the bacteria embedded in a biofilm [30, 31].

Accordingly, we evaluated the effect of curcumin on* S. mutans* biofilm over time. At the SMIC_50_, curcumin was shown to exert both a short-term effect and a long-term effect on the viability of* S. mutant* biofilm, which was also true for 0.12% chlorhexidine. Previous studies showed the effect of chlorhexidine on* S. mutant* biofilm. Yang et al. [32] reported that* S. mutans* biofilm was susceptible to 0.12% chlorhexidine, with a biomass reduction of over 80% observed after treatment with 0.12% chlorhexidine for 1 h. Tamura et al. [33] found that 8 *μ*g/ml chlorhexidine was effective in removing biofilm after 24 h. All of these results illustrate that chlorhexidine has antibiofilm effects, which was consistent with our results. The short-term effect of curcumin on* S. mutans* biofilm also suggested that curcumin has the potential to be a new antibiofilm drug because it exhibited similar effects to chlorhexidine.

*S. mutans* has a regulatory network to integrate its cellular response to environmental change [6]. This study found that, after treatment with curcumin, the amounts of viable bacteria and total bacteria were reduced, as confirmed by the results of CLSM. Because curcumin exhibited no effect on the growth kinetics of* S. mutans*, the reduced quantity of live bacteria indicated a direct effect of curcumin on* S. mutans.*

A biofilm is a highly dynamic and structured community of microbial cells that are enmeshed in an extracellular matrix of polymeric substances such as exopolysaccharides, proteins, and nucleic acids [34]. As reported previously, the production of EPS by* S. mutans *on a surface enhances the local accumulation and clustering of microbes. In addition, as the biofilm develops, the spatial heterogeneities resulting from EPS synthesis form a complex 3D matrix architecture and create environmental and protective niches within the biofilm that can directly modulate caries pathogenesis [34, 35]. Curcumin was shown to destroy the structure of EPS in the short term, decreasing the biomass of EPS, the quantity of total bacteria, and the percentage of lived bacteria. With prolonged curcumin exposure, the structure of the EPS was badly damaged, and the thickness of the biofilm and the number of total bacteria were decreased demonstrably. Khan et al. reported that the extract of* Trachyspermum ammi* seeds could modulate the expression of specific virulence genes by* S. mutans*, which disrupted the accumulation and structural organization of EPS, ultimately affecting the cariogenicity of* S. mutans* [36]. Xu et al. found that the tea catechin epigallocatechin gallate could inhibit the formation of* S. mutans* biofilm by suppressing the gtfs gene [37]. These studies serve as a reminder that the effects of natural products on the EPS of* S. mutans* biofilm are usually exerted by regulating cariogenic genes.

As previously reported, the regulatory systems in* S. mutans,* such as the EPS synthesis system, carbohydrate metabolism system, quorum-sensing system, and two-component transduction system, are connected to the ecological balance and to bacterial carcinogenesis [6, 38]. The results showed that curcumin decreased the expression of cariogenic genes in the plaque of* S. mutans.* Although the majority of the 24 h qPCR results were not statistically significant, the *P* values between the control group and curcumin group were close to 0.05, and the decrease was pronounced. Thus, curcumin has an inhibitory effect on certain genes in* S. mutans* at 24 h.

Gtfs, encoded by the gtfBCD genes, are indispensable for the utilization of glucose and for EPS synthesis in* S. mutans*. They are important factors in biofilm formation and the development of caries [24]. GtfB synthesizes water-insoluble polysaccharide [39], which is the main component of the EPS. Downregulation of the expression of gtfB in curcumin might explain the decreased biomass of EPS in the short term. Oral bacterial aggregation is mediated by interactions between surface-associated glucan-binding proteins (GbpBs) that adhere to glucans, thereby promoting plaque formation [40]. The expression of gbpB was decreased in both treatment groups, which may relate to the reduced thickness of the biofilm. Fructosyltransferase (FTF) is an enzyme that converts sucrose to extracellular homopolymers of fructose, the fructans. FTF is the product of the fruA gene and is an exo-*β*-d-fructosidase that releases fructose from *β*(2.6)- and *β*(2.1)-linked fructans and cleaves fructose from sucrose and raffinose [41–43]. Both ftf and fruA expressions were dysregulated after short-term exposure to curcumin.

In* S. mutans*, the scrA gene in the phosphotransferase system (PTS) encodes a high-affinity permease, which internalizes sucrose. Then, intracellular sucrose-6-PO_4_ is first hydrolyzed by ScrB, a sucrose-6-PO_4_ hydrolase, to produce fructose and glucose-6-PO_4_. In addition, the downstream of scrA and scrB could encode a regulator to change the expression level of scrAB. Both scrAB genes were downregulated in our study, which might contribute to the reduction in the* S. mutans* biofilm. In the oral environment,* S. mutans* is subjected to rapid pH fluctuations arising from the availability of carbohydrates. The atpH gene in* S. mutans* encodes subunit C of a multisubunit enzyme (F1F0-ATPase) involved in intracellular pH regulation and acid tolerance [44, 45]. Gene expression analysis found that atpH was downregulated in* S. mutans* biofilm after treatment with curcumin for 5 min and 24 h. Thus, curcumin inhibits the acid tolerance ability of* S. mutans* and probably decreases the cariogenic traits of* S. mutans.*

The quorum-sensing system is an essential component of entire gene regulation networks responsible for the adaptation of bacteria in biofilms [46]. Two well-studied quorum-sensing systems exist in* S. mutans*: the ComCDE system, which can enable intraspecies cell-cell communication and has been proved to have a positive regulatory effect on the expression of biofilm-related genes such as gtfB, gtfC, and gbpB in* S. mutans*, and the LuxS system, which can catalyse the formation of the signal peptide AI-2 to mediate interspecies and intraspecies interaction in the multispecies plaque community [6, 47]. The findings of the current work suggest that curcumin can potentially contribute to reducing the quantity of lived bacteria in the biofilm and decrease the biomass of EPS, by decreasing the cariogenic potential of* S. mutans* biofilm, via inhibiting the expression of ComCDE system and LuxS system. The short-term inhibitory effect is more significant than the long-term effect. There is a decreasing tendency in the VicR expression of* S. mutans* after curcumin treatment, suggesting that curcumin may act by inhibiting the expression of VicR. The regulatory network of cariogenic genes in* S. mutans *is complex. It was reported that the ComR/ComS is the second quorum-sensing system in* S. mutans* [48]. The cell-to-cell communication system in* S. mutans* is not clear. Further research is required in this field.

*S. mutans* possesses a series of cell-surface proteins such as SpaP (also known as antigen I/II, Pac, P1 and antigen B), which are anchored to the cell envelope by sortase A (SrtA). Sortase A is encoded by the gene srtA and recognizes a motif consisting of leucine, proline, X, threonine, and glycine (LPXTG, where X is any amino acid) [33, 49]. Natural products attract substantial attention because they can inhibit srtA in vitro and in vivo but do not significantly decrease the viability of bacteria. The current study demonstrates that curcumin can inhibit the expression of spaP and srtA with an SMIC_50_ of 500 *μ*M. The present study explored the effect of curcumin on* S. mutans* UA159. However,* S. mutans* has heterogeneity in different strains. Much more strains of* S. mutans* are needed to be test in future.

In conclusion, curcumin has both a short-term and a long-term antibacterial effect. Additionally, it is a food-grade natural product with a similar effect to that of chlorhexidine and it takes effect faster than 0.12% chlorhexidine. Curcumin could be an alternative strategy to treat oral disease. Curcumin is a promising antibacterial agent with potential for wider clinical application in the future.

## Figures and Tables

**Table 1:** Nucleotide sequence of primers used for qRT-PCR.

Gene	Primer sequence (5′-3′)	
	Forward	Reverse
gtfB	ACACTTTCGGGTGGCTTG	GCTTAGATGTCACTTCGGTTG
gtfC	CCAAAATGGTATTATGGCTGTCG	GAGTCTCTATCAAAGTAACGCAGT
gtfD	TTGACGGTGTTCGTGTTGAT	AAAGCGATAGGCGCAGTTTA
fruA	TGTAGGTCTCGGTTTGTGGGAC	TCTTGAGCCAATGCTTCTGGTG
gbpB	AGCAACAGAAGCACAACCATCAG	CCACCATTACCCCAGTAGTTTCC
Ftf	CTGACATAACTACGCCAAAG	TGCTTAAATTAATACCAGCTTC
luxS	CCAGGGACATCTTTCCATGAGAT	ACGGGATGATTGACTGTTCCC
comC	GACTTTAAAGAAATTAAGACTG	AAGCTTGTGTAAAACTTCTGT
comD	CTCTGATTGACCATTCTTCTGG	CATTCTGAGTTTATGCCCCTC
comE	CCTGAAAAGGGCAATCACCAG	GGGGCATAAACTCAGAATGTGTCG
VicR	TGACACGATTACAGCCTTTGATG	CGTCTAGTTCTGGTAACATTAAGTCCAATA
atpH	ACCATACATTTCAGGCTG	TTTTAGCACTTGGGATTG
scrA	GATTGCCCTCAGCAGTTGACAT	GCTGGGAAACTTTGATGGAGAC
scrB	ACAGCCTGTCCTGATTTATAGTC	CTGGTAACCCAATCCATGAGAC
srtA	GAAGCTTCCTGTAATTGGCG	TTCATCGTTCCAGCACCATA
spaP	GACTTTGGTAATGGTTATGCATCAA	TTTGTATCAGCCGGATCAAGTG
16sRNA	CTTACCAGGTCTTGACATCCCG	ACCCAACATCTCACGACACGAG
